# Residues contributing to drug transport by ABCG2 are localised to multiple drug-binding pockets

**DOI:** 10.1042/BCJ20170923

**Published:** 2018-05-04

**Authors:** Megan H. Cox, Parth Kapoor, Deborah A. Briggs, Ian D. Kerr

**Affiliations:** School of Life Sciences, University of Nottingham, Queen's Medical Centre, Nottingham NG7 2UH, U.K.

**Keywords:** ABC transport proteins, ABCG2, asymmetry, binding site, molecular docking, multidrug resistance

## Abstract

Multidrug binding and transport by the ATP-binding cassette transporter ABCG2 is a factor in the clinical resistance to chemotherapy in leukaemia, and a contributory factor to the pharmacokinetic profiles of many other prescribed drugs. Despite its importance, the structural basis of multidrug transport, i.e. the ability to transport multiple distinct chemicals, has remained elusive. Previous research has shown that at least two residues positioned towards the cytoplasmic end of transmembrane helix 3 (TM3) of the transporter play a role in drug transport. We hypothesised that other residues, either in the longitudinal span of TM3, or a perpendicular slice through the intracellular end of other TM helices would also contribute to drug binding and transport by ABCG2. Single-point mutant isoforms of ABCG2 were made at ∼30 positions and were analysed for effects on protein expression, localisation (western blotting, confocal microscopy) and function (flow cytometry) in a mammalian stable cell line expression system. Our data were interpreted in terms of recent structural data on the ABCG protein subfamily and enabled us to propose a surface-binding site for the drug mitoxantrone (MX) as well as a second, buried site for the same drug. Further mutational analysis of residues that spatially separate these two sites prompts us to suggest a molecular and structural pathway for MX transport by ABCG2.

## Introduction

As their name implies, multidrug resistance (MDR) pumps are able to transport a large range of chemically diverse substrates out of cells. While presumably this was driven by the evolutionary advantage of being able to avoid xenobiotic toxic chemicals, it has the unwanted consequence today of contributing to the failure of both antimicrobial chemotherapy and cancer chemotherapy. In addition, it is widely believed that MDR pumps are major contributors to the ADME (absorption, distribution, metabolism, and excretion) profile of most prescribed drugs [1]. In humans, three members of the ATP-binding cassette family of transporters are particularly associated with MDR transport, namely ABCB1 (P-glycoprotein), ABCC1 (multidrug resistance-associated protein 1), and ABCG2 (breast cancer resistance protein) [2,3]. A mechanistic description of the mechanism of these pumps' poly-specificity would be invaluable in efforts to circumvent their unwanted actions.

Intensive structural and functional studies have started to shed light on the mechanisms of both ABCB1 and ABCC1 [4–6] and detailed descriptions of which residues are responsible for forming binding sites for particular transport substrates are starting to emerge [4,7–9]. For ABCG2, the situation is less well resolved [10]. Previous research has established that there are multiple, pharmacologically distinct binding sites on ABCG2 for drugs, whose affinities are altered by nucleotide binding [11,12], according to the similar power stroke model for ABCB1 [13]. The data demonstrated allosteric effects of mitoxantrone (MX), rhodamine 123, and Hoechst 33342 on the binding of [^3^H]-daunorubicin (DNR) indicating that these drugs bind at sites which do not directly overlap with that for DNR. However, it remains to be demonstrated whether these sites are spatially well separated on the protein, or whether they occupy adjacent or overlapping surfaces.

The predominantly hydrophobic nature of ABCG2 drug substrates means that interaction through the transmembrane domains is likely [14], and the existing literature on residues which influence substrate specificity reinforces this. The most well-known residue to be involved in transport of substrates is arginine 482 in transmembrane helix 3 (TM3); indeed, its role in ABCG2 function emerged at the same time as the gene was cloned. Three different groups isolated two variants of the ABCG2 sequence, differing in specificity, and differing in the identity of the amino acid residue at position 482 [15–17], with R482 soon confirmed as the wild-type (WT) sequence [15,17] and R482T identified as a drug selected variant of the protein [16]. This residue has continued to be widely studied, with mutations being shown to confer a broader substrate specificity compared with the WT sequence [18–20]. The adjacent turn of the TM3 α helix contains a conserved proline residue (P485) and we, and others, have mutated this residue, again showing effects on substrate specificity [21,22]. For example, we demonstrated that a mutant P485A was less effective at transporting the porphyrin derivative pheophorbide A (PhA) compared with the WT sequence [21]. With these residues being one turn apart in an α-helix, it seems reasonable to suggest that they would be spatially close. However, it remains difficult to be certain about the structural basis of their contribution to drug binding. Low-resolution electron microscopy of the protein was, until very recently, the only source of structural information we had for the protein [23–25]. Since the commencement of this study, our structural knowledge of ABCG2 has been bolstered by publications of an X-ray crystallographic structure of the related ABCG5/G8 heterodimeric transporter [26], a medium resolution cryoelectron microscopy structure of ABCG2 complexed with an inhibitory antibody [27], and homology models of ABCG2 built from these structural templates [28–30]. Such models provide a valuable framework for data interpretation, but currently do not provide an unambiguous understanding of drug binding and release sites on ABCG2.

For the present study, we considered the two residues in TM3 that have been shown to impact either upon the interaction with the transported substrate, or upon the transmission of conformational changes necessary to support transport, i.e. R482 and P485 located one turn apart in TM3. Given that the TM3 sequence is highly conserved across ABCG2 sequences (Supplementary Figure S1), we decided to investigate residues both N- and C-terminal to R482 to determine if there were other residues within TM3 that could influence the transport of ABCG2 substrates. In other words, we are asking ‘is there a general role for TM3 in the substrate specificity of ABCG2?’

We also reasoned that if a pair of residues in TM3 could influence specificity, then residues in similar positions on neighbouring helices (with respect to the plane of the membrane) could influence transport substrate specificity as well. Such a situation is observed with ABCB1, where lines of evidence have supported the concept of residues on multiple adjacent α-helices contributing to drug binding and transport [7–9,31,32]. This hypothesis led us to mutate residues in TM1-2 and TM4-6 that would be at a similar position (a ‘lateral slice’) in the membrane as P485 and R482.

In total, we made ∼30 single amino acid mutations to alanine, and these were examined for their effects on membrane targeting and protein function, using three substrates of either WT or R482G-mutated ABCG2 to ensure that we captured as much pharmacological data as possible. The data are interpreted in terms of recent structural models for ABCG2 and contribute to what is now a rapidly emerging picture of ABCG2:substrate interaction.

## Methods

### Mutagenesis

All single mutations were made in a vector (p3.1zeo_sfGFP_ABCG2) which encodes an N-terminal superfolder GFP (sfGFP)-tagged ABCG2 as previously described [33]. Mutations were introduced using oligonucleotide-directed site-directed mutagenesis using QuikChange-like technology (using primers listed in Supplementary Table S1), and either Pfu Polymerase (Promega) or Phusion DNA polymerase (NEB). Following DpnI digestion and transformation, putative mutant plasmids were obtained from overnight 5 ml bacterial cultures using a commercial plasmid preparation kit (Qiagen or Machery-Nagel). All mutant plasmids were confirmed by DNA sequencing across the entire sfGFP-ABCG2 cDNA (Source Bioscience).

### Cell culture

Unless stated otherwise, human embryonic kidney cells (HEK293T) were maintained in T25 flasks (Corning) at 37°C, 5% CO_2_ in Dulbecco's Modified Eagle Medium (DMEM, 4500 mg l-glucose, l-glutamine, sodium pyruvate, and sodium bicarbonate) supplemented with 10% (v/v) foetal calf serum (FCS; Gibco), 100 units/ml penicillin and 100 µg/ml streptomycin (Invitrogen). The cells were routinely monitored for their morphology and confluency and at 80–90% confluency the medium was removed and the cells were washed once with 2 ml of sterile phosphate-buffered saline (PBS) and then incubated (37°C) with 0.3 ml of trypsin/EDTA (Invitrogen) for 1–2 min to detach cells from the growing surface. Cells were then resuspended in DMEM by repeated pipetting and pelleted at 500 ***g*** for 5 min to remove excess trypsin. Pelleted cells were resuspended in the medium and re-plated typically at a 1:10 dilution of the original culture.

### Transfection and selection of stable cell lines

Cells were seeded at 2.5–3 × 10^5^ cells/well into a 6-well plate 24 h prior to transfection. Three hours prior to transfection, the medium was replaced with DMEM supplemented with 5% (v/v) FCS. Cells were transfected using linear polyethyleneimine (PEI; Polysciences Inc.) at a molar PEI nitrogen: DNA phosphorous ratio of 15:1, by adding preformed PEI/DNA complexes dropwise to the growth medium [34]. Successful transfection was confirmed 24 h later using an inverted epifluorescence microscope (Hg lamp, Carl Zeiss) and the medium was then replaced with DMEM supplemented with 10% (v/v) FCS. A further 24 h later, cells were detached (by trypsinisation) and transferred to T25 flasks with a fresh medium supplemented with 200 µg/ml Zeocin (ThermoFisherScientific) for a period of 2–3 weeks with periodic media changes until death of the non-transfected cells was observed and Zeocin resistant colonies of transfected cells had developed. Once healthy colonies were obtained, the cells were maintained at a lower Zeocin concentration (40 µg/ml).

### SDS–PAGE and western blotting

Cells were harvested by centrifugation (1500 ***g***, 4°C, 5–10 min) and cell pellets were then resuspended in ice-cold PBS supplemented with 10% v/v glycerol, before being recentrifuged to remove any remaining medium. Cell pellets were then lysed by sonication in ice-cold PBS/glycerol by 3 × 10 s bursts at 40% power (Microsonics). Insoluble cell debris was removed by brief centrifugation and protein concentration determined by a commercial Lowry assay (Bio-Rad DC). Protein was resolved on 8 or 10% w/v polyacrylamide gels [35] and stained with InstantBlue (Expedeon). For western blotting, proteins were electroblotted onto nitrocellulose, blocked by incubation in blocking buffer (PBS supplemented with 0.1%v/v Tween-20 and 5%w/v non-fat milk) and then incubated with anti-ABCG2 monoclonal BXP-21 antibodies (Merck Biosciences) at 1:1000 in blocking buffer at 4°C overnight. The nitrocellulose was then washed with PBS/Tween and incubated for 1 h at room temperature with horseradish peroxidase conjugated secondary antibody (1:2000, DAKO). Following washing, the specific proteins were detected using a SuperSignal® West Pico Chemiluminescent Substrate (ThermoFisherScientific).

### Cell imaging

Live cell imaging of HEK293T cells stably transfected with sfGFP_ABCG2 mutant isoforms was performed either with an ImageXpress (IX) Ultra confocal plate reader (Molecular Devices), using a plan-apochromat 40× objective, with excitation wavelength of 488 nm and emission bandpass filter of 525/50 nm, or with a LSM710 confocal laser scanning microscope (Zeiss), using a plan-apochromat 63×/1.40 Oil Ph3 DIC M27 objective and 2% argon laser, with excitation wavelength of 488 nm and emission collected at 500–550 nm. For confocal plate reader analysis, cells were seeded in poly-l-lysine-coated, 96-well black-walled, clear-bottom plates (Greiner) at a cell density of 3 × 10^4^ cells/well in DMEM 24 h before imaging. For confocal microscopy, cells were seeded at 2.5 × 10^5^ cells/well in 35 mm glass bottom dishes (MatTek Corp®) 24 h prior to imaging. In both cases, cells were subsequently washed twice with pre-warmed (37°C) phenol-red free HBSS (Hank's Balanced Salt Solution, Sigma–Aldrich) immediately prior to imaging.

### Drug transport analysis

To evaluate the functionality of mutant ABCG2 isoforms in HEK293T cell lines, drug accumulation assays were performed by flow cytometry. All drugs, solvents, and inhibitors were from Sigma–Aldrich. Cells were seeded at 1 × 10^6^ cells/ml in phenol-red free DMEM and incubated with either DMSO (solvent control 0.2% v/v), MX (10 µM), PhA (10 µM), or DNR (10 µM) in the presence or absence of the ABCG2 inhibitor Ko143 (1 µM, [36]) at 37°C for 30 min with occasional agitation. Cells were centrifuged to remove excess drug at 350 ***g***, 4°C for 5 min prior to a second incubation at 37°C for 60 min with either phenol-red free DMEM only or phenol-red free DMEM plus Ko143 (for samples originally incubated with drug + inhibitor). Cells were centrifuged as above and then resuspended in phenol-red free DMEM prior to the analysis by flow cytometry. GFP fluorescence was determined using excitation wavelength 488 nm and emission at 526 nm, MX fluorescence was measured using excitation at 635 nm and emission at 670 nm using FC500 flow cytometer (Beckman Coulter). PhA fluorescence was measured using excitation at 355 nm and emission at 692 nm and DNR fluorescence was measured using excitation at 490 nm and emission at 630 nm, respectively, using Moflo Astrios flow cytometer system (Beckman Coulter). DNR fluorescence was separated from GFP fluorescence during data acquisition by compensation.

Data were analysed using Kaluza analysis version 1.5 (Beckman Coulter). Cells were gated based on size, dispersity, and fluorescence. A GFP profile demonstrating a clear split population for GFP fluorescence enabled further gating of populations with lower and higher expression of sfGFP-ABCG2; it was found that there was no efflux from the lower expressing population, so this was excluded from further analysis. The vehicle control (autofluorescence) was used as a baseline and therefore the median fluorescence of vehicle-treated cells was subtracted from the values for drug with or without inhibitor. The fractional difference between the sample with drug plus inhibitor and drug alone was then calculated. This represents the relative efflux of an ABCG2 isoform for a particular drug, and this was finally corrected for the expression level of ABCG2 isoforms compared with WT GFP-ABCG2. The normalised fractional values were analysed using GraphPad Prism and were subjected to one-way ANOVA with a Dunnett's multiple comparisons against WT ABCG2 to determine if any of the mutations differed in their ability to efflux drug.

### Cell surface expression analysis

Cells were seeded at a cell density of 1 × 10^6^ cells/ml in a blocking buffer (PBS containing 1% w/v BSA) and incubated with primary monoclonal antibody anti-ABCG2, clone 5D3 (1:200; Millipore), or an isotype control. Cells were incubated on ice for 30 min and subsequently washed by two cycles of pelleting (350 ***g***, 5 min, 4°C) and resuspension in the blocking buffer. Cells were then incubated with secondary antibody AlexaFluor647 (AF647; 1:200 Thermofisher Scientific) on ice for 60 min, followed by the same washing steps. Finally, the cells were resuspended in the blocking buffer and analysed for GFP (488/526 nm) and AF647 (650/670 nm) using an Astrios cytometer (Beckman Coulter). Data were analysed using Kaluza, with prior gating for size and dispersity.

### Molecular visualisation and drug docking

All molecular docking studies were performed using a model for ABCG2 based upon the structure of the homologous ABCG5/G8 structure [26], provided by Dr Thomas Stockner [29]. To enable the docking, co-ordinates and parameters for MX were obtained from ChemSpider and the protein and MX (an ABCG2 substrate) were converted to PDBQT (Vina executable) files, via AutoDockTools (v1.5.6). A grid box centred on the approximate geometric midpoint of the TMD of an ABCG2 monomer (residues 517/518) with dimensions 36 × 20 × 20 (Å) was assigned as the search region for AutoDock Vina ([37], The Scripps Research Institute) molecular docking software. The exhaustiveness of the search was manually set to 128, all other parameters being the default, to find the most energetically favourable poise. Visual inspection of the PDBQT output files was accomplished using PyMOL© (Schrodinger, LLC).

### Data analysis

Other numerical analyses were performed using Microsoft Excel and statistical analysis was performed using GraphPad Prism. All the statistical tests were analysed with *P*-values less than 0.05 considered significant for a given set of data.

## Results

### Identification of residues for mutation

**Figure 1.**
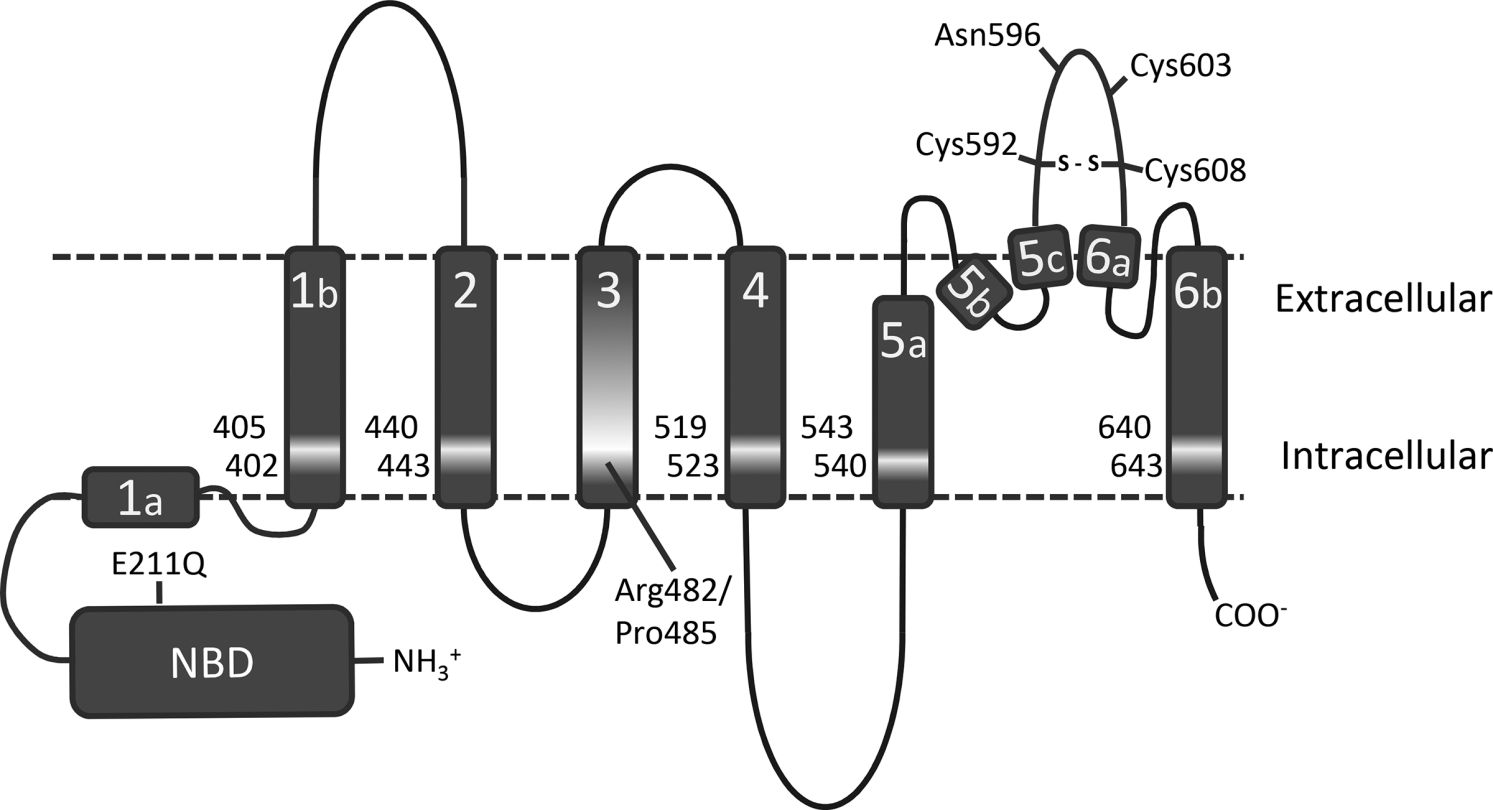
Location of residues for mutation in ABCG2. A cartoon representation of the structural topology of ABCG2 [27] is shown with the TM helices represented as grey rectangles. The location of residues for mutation is indicated by lighter shading together with the single amino acid positions. Several other residues known to be important for intra- and inter-molecular disulfide bond formation (C592, 603, and 608) and glycosylation (N596) are marked for ease of reference, as is the Walker B glutamate mutation E211Q used as a catalytically inactive mutant control.

We and others are interested in how MDR pumps of the ABC transporter family are able to interact with and transport a diverse range of chemicals out of cells. For ABCG2, there have been reports identifying particular residues that influence substrate specificity, the most notable of which would be R482 and P485 in TM3 [20,21]. This led us to consider whether the highly conserved TM helix 3 itself could harbour further residues of interest, with respect to substrate specificity, and thus we mutated a series of residues predicted by transmembrane topology mapping to be located in TM3 (residues 477–497; Supplementary Table S1 and Figure S1). We also considered whether residues in other TM regions, which might be at a similar position to P485 and R482 (with respect to the plane of the membrane), could also be implicated in substrate specificity, leading us to mutate a series of residues in TM1, TM2, TM4, TM5, and TM6, which we refer to as ‘lateral slice’ residues. In each case, we mutated residues to alanine, as this residue is structurally neutral with respect to the torsional requirements for an α-helix, and is thus is unlikely to see any significant perturbation to overall protein folding [38]. The schematic positions of the residues mapped onto a topology model of ABCG2 are shown in Figure 1.

We constructed all mutations in an isoform of ABCG2 which bears an N-terminal sfGFP tag, which we have previously demonstrated to retain functionality and localisation in HEK293T cells [21,33]. Following mutagenesis, we selected stably transfected HEK293T cell lines using Zeocin. Previously constructed WT, and catalytically inactive, E211Q, isoforms were utilised in the study as positive and negative control comparisons, respectively.

**Figure 2.**
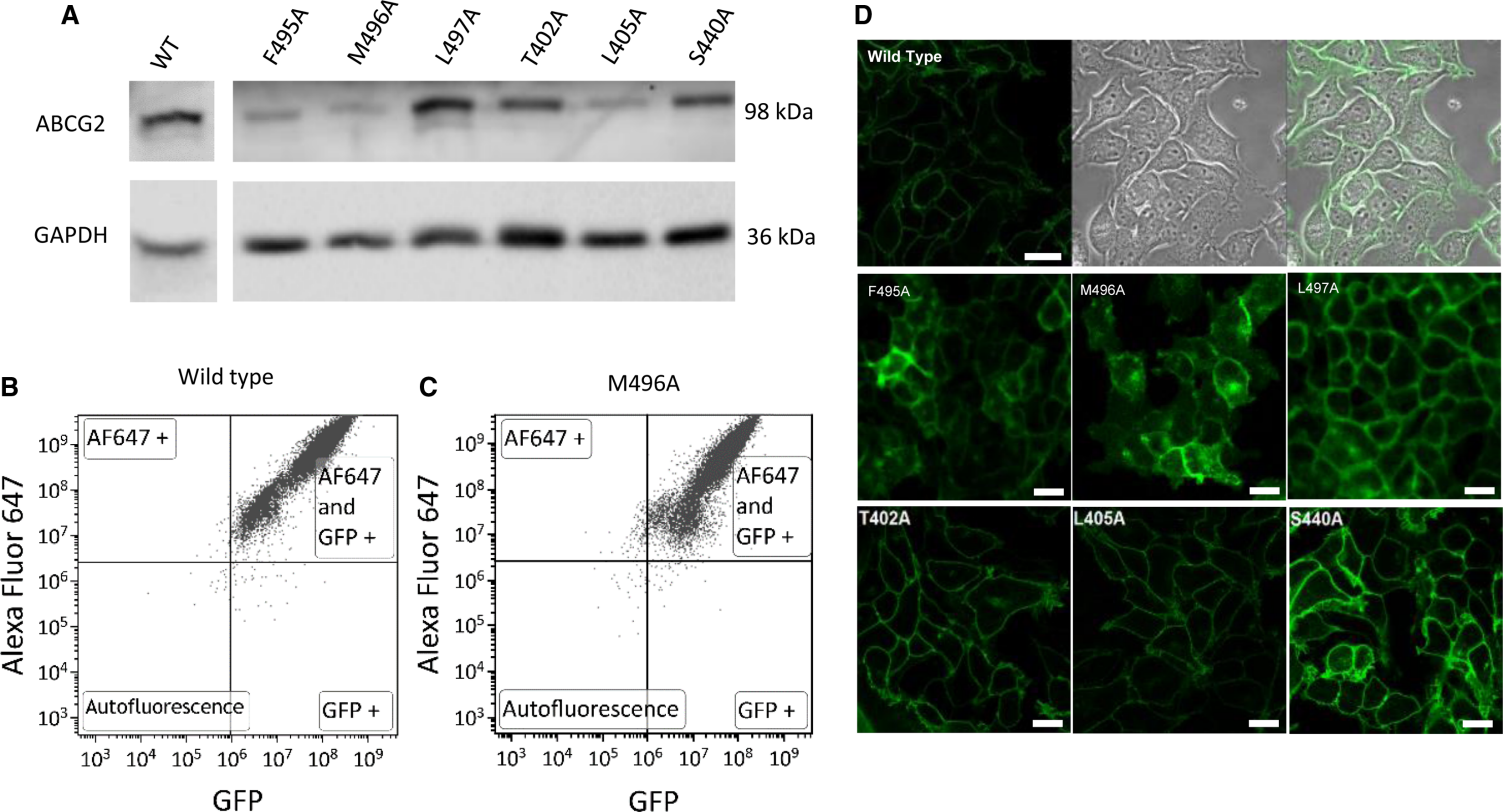
Cell surface localisation of the ABCG2 variants. (**A**) Western blotting of cell lysates was performed with antibodies specific for ABCG2 (90–100 kDa due to the presence of the GFP tag) or GAPDH (35–40 kDa). The vertical break in the figure indicates removal of other lanes such that (**A**) and (**D**) show the same mutants. (**B** and **C**) Cells were labelled with anti-5D3 antibody and a secondary antibody conjugated to AlexaFluor 647 enabling individual cells to be quantified for surface expression (*y*-axis) and GFP expression (*x*-axis). The upper right quadrant represents cells positive for cell surface expression isoforms and indicates over 90% of the cells for the two isoforms show surface expression of sfGF_ABCG2. (**D**) Cells were imaged using a LSM710 confocal laser scanning microscope (Zeiss, using a plan-apochromat 63×/1.40 Oil Ph3 DIC M27 objective and 2% power argon laser, with excitation wavelength of 488 nm and emission collected at 500–550 nm). For the WT cells, three panels are presented the fluorescence (left), bright field (middle) and merged data (right). For the variants, only the fluorescence image is shown. Scale bar represents 20 µm. All other mutants not shown here are in Supplementary Figure S2.

Some mutant isoforms of ABC transporters (including the gout-associated Q141K mutation in ABCG2 [39] and a mutation that we have previously characterised in extracellular loop 3; I573A [21]) are improperly folded and fail to be localised to the plasma membrane. To screen for any of our mutants being associated with a trafficking defect, we verified protein expression and trafficking with three complementary approaches (Figure 2). Firstly, we performed western blotting of whole cell lysates; although there was some variation in the ABCG2 expression level across the mutant isoforms analysed, all isoforms were expressed as fully glycosylated proteins (Figure 2A for a selection of the mutations). To confirm that this reflected just heterogeneous expression levels within stable cell lines and was not a reflection of trafficking defects, we performed a 2-channel flow cytometry assay that measured GFP fluorescence as a measure of total ABCG2 expression, and immuno-detection of a surface-exposed epitope (5D3, [40]) in intact cells as a measure of plasma membrane expression of ABCG2. In all variants examined, the percentage of cells positive for both GFP expression and 5D3 reactivity exceeded 90% suggesting that sfGFP-ABCG2 variants were correctly localised to the plasma membrane (Figure 2B,C). Finally, we further utilised the sfGFP tag to demonstrate via confocal microscopy that all mutant isoforms of ABCG2 were reaching the cell surface, with the overwhelming majority of the GFP signal observed at the plasma membrane (Figure 2D and Supplementary Figure S2). This allows us to infer that the introduction of the point mutations was not deleterious to the folding and trafficking of ABCG2.

**Figure 3.**
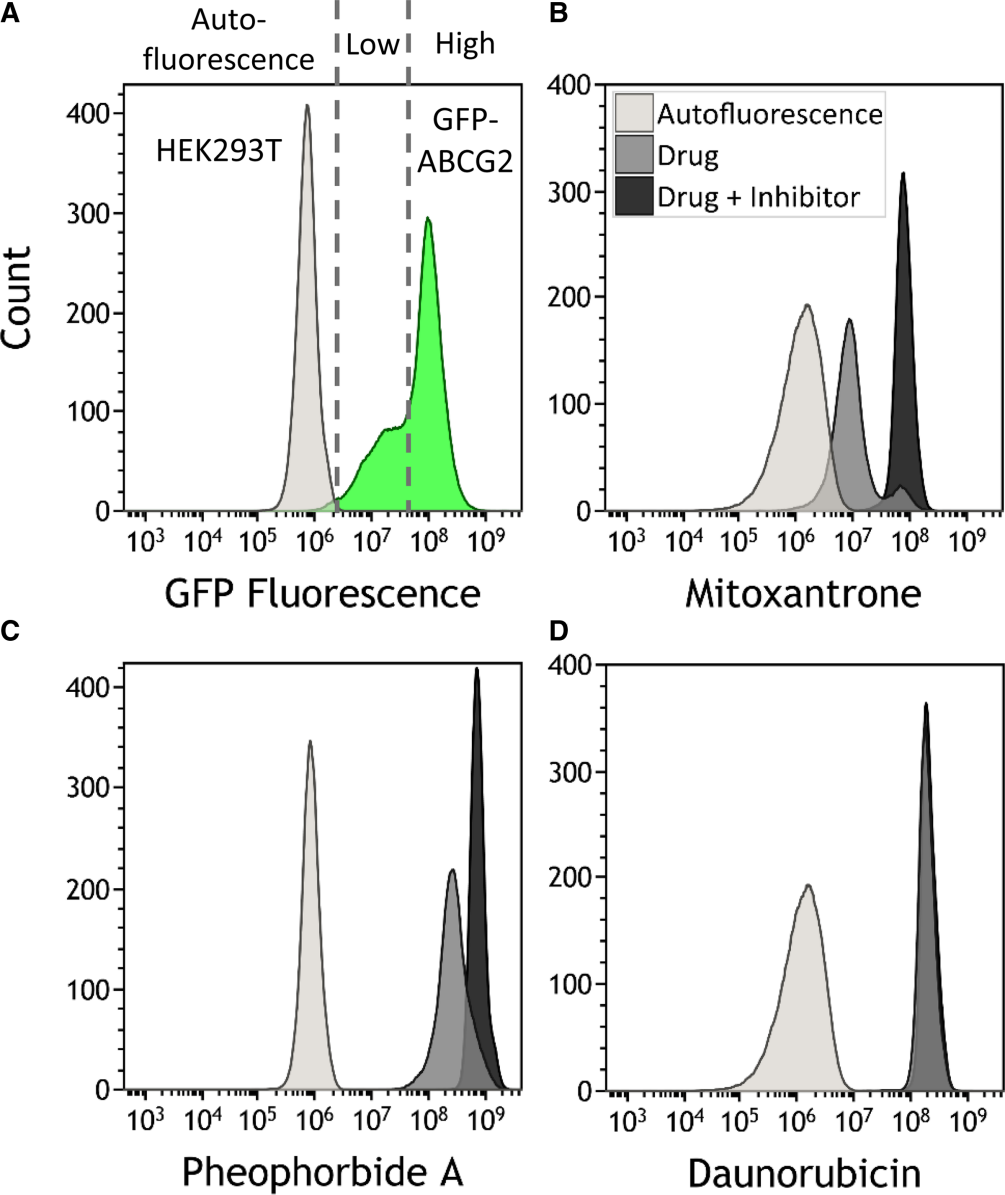
Functional analysis of ABCG2 variants. (**A**) Cell populations were gated for GFP fluorescence (i.e. high versus low expression cells (green shading)), and subsequently this gate used to analyse MX accumulation. For individual drugs (**B**–**D**), data are then presented as control cells' autofluorescence (light grey distribution), drug alone (medium grey) and drug + inhibitor (dark grey). Cell populations were analysed by flow cytometry, using excitation and emission wavelength pairs GFP 488/526 nm; MX 635/670 nm; PhA 355/692 nm and DNR 490/630 nm. At least 10 000 events were analysed for each data set, and the data are representative of at least three independent experiments.

**Figure 4.**
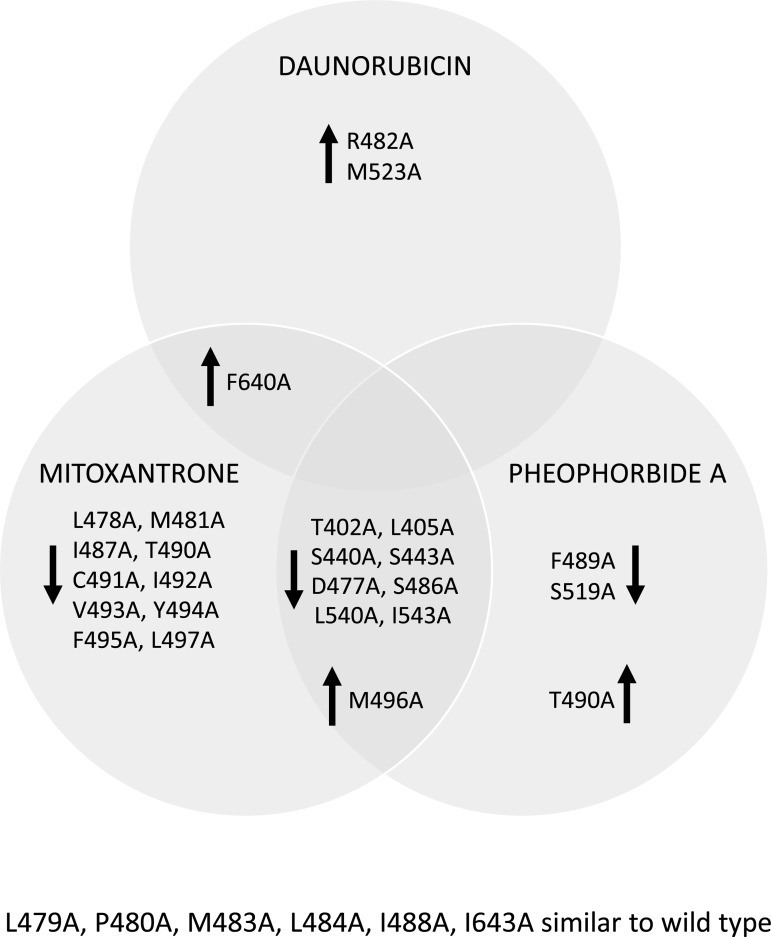
Functional effects of ABCG2 variants. ABCG2 mutant isoforms were analysed for drug export function as shown in Figure 3, and are summarised as either enhanced (upward arrow) or reduced (downward arrow) Ko143-inhibited transport, compared with the WT isoform, with *P* < 0.05 judged as statistically significant. As indicated in Figure 3 all experiments were repeated on at least three independent occasions.

Having demonstrated that the mutants do not impair trafficking of ABCG2, we wished to determine whether any of the residues are involved in the transport of ABCG2 substrates. Flow cytometry provides a reliable method to obtain this information [41]. We studied the transport of two known substrates of WT ABCG2, namely MX and PhA [21], as well as DNR, which is a substrate of the well-studied mutant ABCG2 isoform, R482G, but not of the WT protein [42]. In all cases, cells and fluorescent drug substrates were incubated in the presence or absence of the ABCG2-specific inhibitor Ko143 [36], to ensure that ABCG2-dependent transport is being quantified. Typical data sets are presented in Figure 3; for some mutants, our mixed-expressing cell lines presented two distinct populations with regard to sfGFP-ABCG2 expression, with a proportion of the cells showing very little sfGFP-ABCG2 expression (shoulder in the distribution, Figure 3A). Gating using the fluorescence of the GFP tag enabled us to determine the accumulation of substrates in cells expressing a similar level of ABCG2 across the whole range of mutant isoforms, i.e. enabling us to determine that differences in drug transport are not simply the result of different expression levels (Supplementary Figure S3). Incubation in the presence of fluorescent substrate resulted in a population of cells showing an equilibrium level of substrate accumulation (mid-grey populations in Figure 3B–D), well separated from cell autofluorescence (light grey, Figure 3B–D). Inhibition of ABCG2 by Ko143 resulted in increased accumulation of MX and PhA (dark grey populations, Figure 3B,C) with no change observed for the DNR in the case of the WT protein (Figure 3D). Quantitative analysis of the Ko143-dependent rightward shift in cellular fluorescence data (described in the Methods) enabled us to determine the isoforms associated with altered transport of the three tested compounds (Table 1 and Figure 4).

**Table 1:** Ko143-inhibited transport of fluorescent drugs. All data are presented as the mean and standard error of the fractional difference between the sample with drug plus inhibitor compared with drug. The data for MX and PhA were then subjected to one-way ANOVA with a Dunnett's multiple comparisons against the WT. For the DNR data, the positive skew caused by the R482A mutation led us to perform a log transformation of the data shown prior to analysis of significance.

	MX		PhA		DNR	
	Mean	s.e.m.	Mean	s.e.m.	Mean	s.e.m.
HEK293T	0.21	0.05	0.01	0.03	0.11	0.05
WT	6.01	0.81	2.57	0.33	0.37	0.05
E211Q	0.46**	0.16	0.01**	0.08	0.28	0.14
T402A	2.02**	0.29	0.48**	0.02	0.04	0.02
L405A	1.16**	0.21	0.07**	0.00	0.03	0.01
S440A	2.29**	0.35	0.49**	0.02	0.06	0.01
S443A	3.08*	0.48	0.83**	0.13	0.52	0.05
D477A	1.52**	0.46	0.20**	0.10	0.21	0.20
L478A	2.86**	0.08	2.21	0.26	0.38	0.21
L479A	5.70	0.45	3.62	0.60	0.44	0.08
P480A	4.05	0.57	2.31	0.10	0.92	0.39
M481A	3.45*	0.59	2.85	0.59	0.26	0.16
R482A	5.95	0.85	2.42	0.11	8.11**	1.79
M483A	6.74	0.23	1.61	0.44	0.49	0.08
L484A	5.06	0.46	3.51	0.28	0.36	0.14
S486A	3.25*	0.49	1.21*	0.21	0.19	0.08
I487A	2.98**	0.18	1.55	0.23	0.12	0.05
I488A	3.98	0.57	1.80	0.32	0.30	0.09
F489A	3.90	0.63	1.23*	0.09	0.06	0.01
T490A	3.19*	0.32	4.27**	0.43	0.01	0.07
C491A	1.74**	0.17	1.67	0.21	0.26	0.10
I492A	2.90**	0.10	3.68	0.47	0.30	0.21
V493A	1.98**	0.13	1.73	0.18	0.38	0.07
Y494A	2.97**	0.13	2.68	0.68	0.33	0.14
F495A	2.85**	0.32	3.38	0.61	0.35	0.05
M496A	17.28**	1.63	3.92*	0.76	0.87	0.09
L497A	2.61**	0.37	1.50	0.14	0.45	0.25
S519A	4.27	0.65	1.11**	0.09	0.31	0.06
M523A	4.80	0.29	1.59	0.20	1.27**	0.04
L540A	2.57**	0.25	0.94**	0.13	0.09	0.04
I543A	2.03**	0.17	0.41**	0.05	0.09	0.04
F640A	9.26**	0.81	2.11	0.46	1.66**	0.20
I643A	4.06	0.51	1.66	0.21	0.47	0.12

*P < 0.05 and **P < 0.01.

For MX, the WT isoform displayed a 6.01 (±0.81)-fold change in accumulation of drug upon the addition of Ko143. As expected, the catalytically inactive isoform E211Q (which has a charge neutralising mutation in the Walker B motif [43]) shows massively reduced Ko143-inhibited MX export (0.46 ± 0.16). Our mutations showed three phenotypes for MX transport. Firstly, there were a group of mutations where the Ko143-inhibited efflux was not statistically significant from WT (ANOVA with Dunnett's post-test, *P* < 0.05). Secondly, there were 18 mutations where the transport of MX was significantly lower compared with WT. These included modest, but statistically significant reductions, such as for M481A which has a fold change of 3.45 (±0.59), as well as mutations with much larger effects such as L405A which was statistically indistinguishable from E211Q for MX transport. Two further mutations (M496A in TM3 and F640A in TM6) displayed greater Ko143-inhibited MX transport, indicating that both mutations enhance the ability of ABCG2 to transport MX.

Transport of the protoporphyrin derivative PhA was observed for the WT isoform (2.57 ± 0.33-fold increase in accumulation in the presence of Ko143; Table 1). As with MX transport, there were mutations which showed both reductions in pheophorbide transport, and mutations which showed increases in PhA transport. While some isoforms show the same effect on PhA transport as they do on MX transport (e.g. T402A, L405A, S440A and 5 others all show reductions in Ko143-inhibitable transport of MX and PhA), other residues show drug-specific effects. For example, F489A and S519A both reduced the Ko143-inhibited efflux of PhA, but had no effect on MX transport, whereas mutation of T490 to alanine saw a different effect, i.e. a reduction in the transport of MX, but an increase in PhA transport.

For DNR, we confirmed previous observations showing that replacement of arginine at position 482 by a smaller residue results in DNR transport [16,18–20]. In our hands, R482A showed an 8.11(±1.79)-fold increase in accumulation of DNM in the presence of Ko143 compared with the WT protein (0.37 ± 0.05). Mutation of F640A in TM6 and of M523 in TM5 also resulted in significant Ko143-inhibitable DNR efflux (though not as great as the R482A effect) suggesting that these two residues may have impact upon a DNR binding or transport pathway (Figure 4; Table 1). To confirm that effects on drug transport are still dependent on ATP hydrolysis, we made a double mutant of F640A with E211Q (the Walker B catalytic inactivation [43]) and observed complete inhibition of MX transport (Supplementary Figure S4).

Data regarding the effects of mutation on the transport of multiple ABCG2 substrates can be rationalised against structural data to provide a molecular explanation for the effects observed. Recently, many structural models of ABCG2 have become available based upon either homology to the ABCG5/G8 crystal structure, or determined directly from cryo-EM data [27–30]. We performed docking studies of MX onto the homology model of ABCG2 described by Stockner and Kuchler [29], preferring this to the cryo-EM structure [27] for two reasons. Firstly, the ABCG2 structure was obtained in the presence of inhibitory antibodies which lock the transporter in an inactive conformation of unclear significance in the alternating catalytic site model of ABC transporters. Secondly, the resolution of the ABCG2 structure is rather variable and the final structure is partially based on homology to the ABCG5/G8 structure [27].

**Figure 5.**
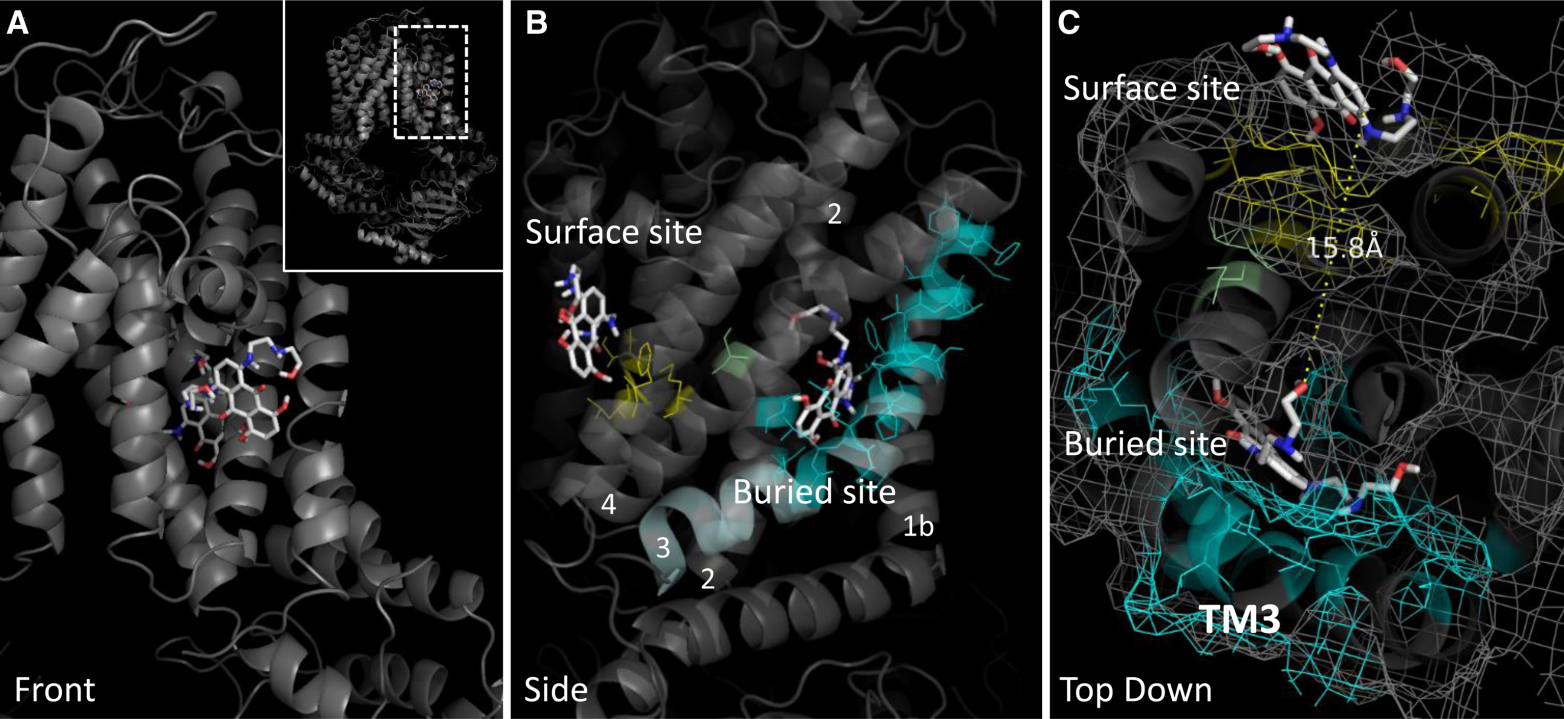
ABCG2 mutants interpreted using a multiple drug-binding site model. ABCG2 modelling and data interpretation was based upon the model for ABCG2 [29] reflecting homology to the heterodimeric transporter ABCG5/G8 [26], shown in a cartoon and surface mesh representation. (**A**) A surface-exposed MX docking site (MX shown in stick format) was identified by AutoDock Vina shown in this view at the front of the model. The second buried site (MX shown in stick format) is at the rear of the view shown. (**B**) An orthogonal view shows the two sites more clearly with residues in cyan indicating those in the buried site that affect MX transport when mutated to alanine, and those in yellow indicating residues in the surface site that affect MX transport when mutated to alanine. (**C**) The third orthogonal view shows both the surface and buried site, separated by 15 Å with one of our residues, S519 shown in green, lying in this hypothetical translocation cavity.

Putative binding sites for MX were observed in two distinct areas of the TMD, with partial similarity to other studies of ABCG2:drug docking (see discussion and Supplementary Table S2 for details [28]). A ‘surface site’ was identified, exposed at the inner leaflet of the membrane (Figure 5A–C) which includes many residues in our lateral slice (M523, L540, I543, F640; shown in yellow in Figure 5B,C) which when mutated to alanine show either a reduction in MX transport, and/or effects on DNR/PhA transport. A second, more ‘buried site’ for MX docking, is also predicted by docking studies with significant contribution from TM helix 3, and also interactions with TM helix 1b, 2 and 4 (Figure 5B) where several more of our function-perturbing residues are located (cyan residues Figure 5B,C; Table 1), including residues in TM3 and the ‘lateral slice’.

**Figure 6.**
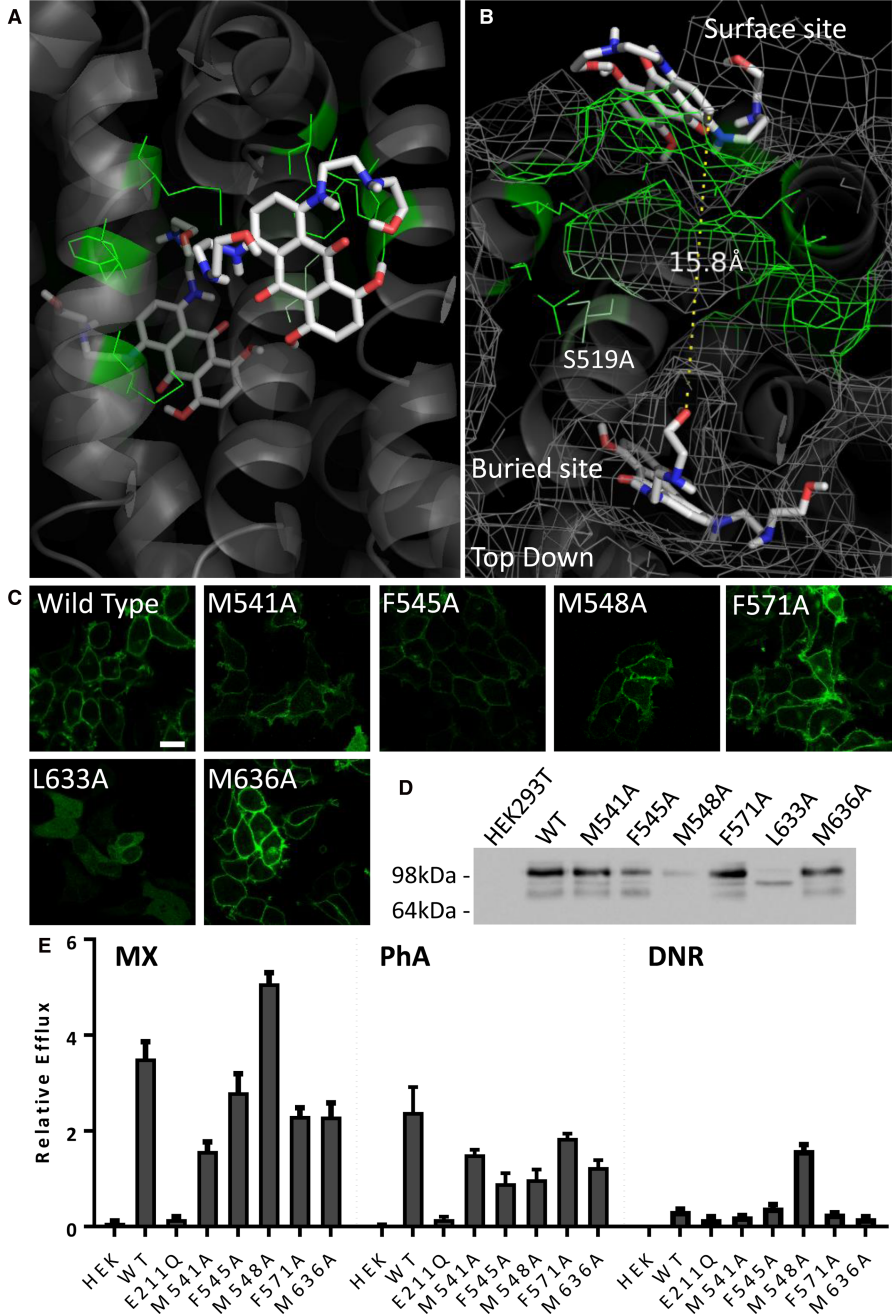
Mutation of a predicted translocation cavity in ABCG2. (**A** and **B**) An additional set of residues (green) were mutated to alanine based upon their localisation to the proposed translocation cavity between the surface and buried MX binding sites. (**C** and **D**) Mutants were sfGFP-tagged and expressed in HEK293T cells, with all bar L633A forming mature protein at the plasma membrane. (**E**) Functional analysis of the mutants demonstrated an enhanced transport of DNR in M548A, and altered MX transport for M541A and F571A and M636A. All data result from at least three independent experiments with error bars denoting standard error of the mean.

Thus, our data have revealed two, spatially distinct, clusters of residues that might have impact on drug binding in ABCG2. Intriguingly, closer inspection of a top view of both sites (Figure 5C) demonstrated that the buried site and the surface site are only 15 Å apart with a narrow sidechain-lined cavity between them. This cavity was lined by one of our lateral slice residues, S519, which has functional effects when mutated (Figure 5B,C, green sticks). This led us to speculate that both sites might be part of an overall translocation pathway for MX. We, therefore, mutated a further selection of six residues in this hypothetical translocation pathway to alanine (green sticks, Figure 6A,B) and established GFP-ABCG2 expressing cell lines (Figure 6C). One of the new six isoforms (L633A) was unable to be trafficked to the plasma membrane and showed an immature band on a western blot (Figure 6C,D) and so its function was not investigated further (although this residue is discussed later). The other five residues were examined for drug export function using our flow cytometric assay (Figure 6E). M548A showed an enhanced transport of MX, and also gained DNR transport function, but significantly lower PhA transport; the other residues predominantly caused a reduction in both PhA transport and MX transport, with no effect on DNR.

## Discussion

ABCG2 is one of three human multidrug pumps whose ability to transport diverse chemicals out of cells implicates it in chemotherapy resistance and drug pharmacokinetics. We wanted to increase the knowledge of how, at a protein structural level, ABCG2 is able to transport many different drugs. We formulated a hypothesis based upon previous data in the literature that had identified residues 482 and 485 in TM3 as being contributors to ABCG2 specificity and drug transport [20–22]. We mutated residues throughout the whole of TM3, on both the cytoplasmic and extracellular side of R482/P485, and also mutated residues in a lateral slice that would encompass pairs of residues in TM1, 2, 4, 5, 6 at approximately the same height in the membrane as R482/P485 (all mutations are shown on a structural model of ABCG2 in Supplementary Figure S5). Residues were mutated to alanine as this has a high propensity to adapt an α-helical conformation [38] (and our residues all reside in TM helices). None of our initial mutations dramatically altered protein targeting to the plasma membrane, enabling us to observe effects on the transport of the WT substrates MX and PhA, and the non-WT substrate DNR. The fact that only a very few of our mutations affected DNR transport suggests that the recognition and transport pathway for this drug is distinct from that for MX and PhA. By extension, the recognition and transport for MX and PhA may share structural features, as we have identified many mutations with similar effects on their transport.

Our residue choice was extensive and so there is some overlap with published data for ABCG2. Our data are consistent with previous studies on R482 (summarised in [44]) and further cement the importance of this residue in the function of ABCG2 [20], agreeing with the interpretation of Hegedus and colleagues that mutation of 482 has effects on local conformation and dynamics that manifest as changes in selectivity [28]. Three other TM3 residues have been mutated; C491 has been studied by groups trying to identify residues involved in disulfide bond formation in ABCG2, and whether the generation of a Cys-less ABCG2 is feasible [45,46]. Cys-491 mutation to alanine was well tolerated in an isoform with multiple Cys → Ala replacements (although it was not studied as a single amino acid mutation) in one study, whereas mutation to serine resulted in an irinotecan sensitivity phenotype indicating that this residue may have a mild effect on drug transport [45,46]. In the present study, we showed a modest reduction in MX transport (Table 1) which is not dissimilar to these previous studies. Residue 489 in TM3 is the site of a common polymorphism (Phe to Leu); studies of this isoform expressed heterologously in insect cells are consistent with our data on the F498A mutation in mammalian cells showing that there is a significant reduction in PhA transport, and possibly implicating aromaticity of position 489 as crucial for PhA transport [47]. Mao and colleagues have previously mutated all prolines in the ABCG2 TM regions but they, and us, find no defects in transport resulting from a P480A mutation [22]. In our choice of residues in the ‘lateral slice’, only T402 has previously been studied. Mutation to alanine had a generally reductive effect on drug transport in accordance with the data presented here [48].

Our data (summarised in Figure 4) show that many single alanine mutant isoforms of ABCG2 showed significant changes to the transport of MX. Interestingly, structures of other targets for MX (topoisomerase II and serine kinases) show that it can interact with different proteins through different binding interactions and conformations [49–51], suggesting that there may be multiple interaction sites for MX on ABCG2. We used extensive molecular docking to investigate possible interaction sites for MX within the TMD region of a recently published model of ABCG2 [29] and identified two distinct sites. Across the two sites, there were 20 different poises (16 in the buried site, 4 in the surface site), but no other sites were predicted to bind MX. Mutation of residues located in a cavity separating these two sites also resulted in effects on MX transport which leads us to believe that we have defined part of a transport pathway for MX (see below).

It would be of interest to extend our docking studies to other drugs and to look at reconciling the functional effects we observe with mutated ABCG2 isoforms *in vitro* with the same mutations made *in silico*. This would require solvating the ABCG2 model in a representative lipid bilayer, and would also require us to generate equivalent models of the single alanine mutations prior to any docking studies. Such work is beyond the scope of the current investigation, but our docking with MX *in vacuo* does permit comparison to other recent studies describing MX binding sites in ABCG2 [28–30]. In their homology modelling paper on ABCG2, László et al. describe four possible binding sites, two of which (referred to as site 2 and 3 in [28]) contain several of our investigated residues. For ease of comparison, we present the residues in Hegedus' sites 2 and 3 with our proposed binding site residues in Supplementary Table S2. Site 2 has contributions from TM1, TM3, and TM4 and is lined by, inter alia, T402, L405, S440, S443, D477, L478, M481, R482, P485, and S486. Remarkably, all but two of these residues show a dual effect in reducing the ability of ABCG2 to efflux both MX and PhA, and the other two affect MX transport only (L478 and M481) [28]. In their modelling study, Ferreria et al. propose a possibility that both MX and cholesterol can interact at an extracellular surface groove, part of which is localised close to the surface site identified here. Interestingly, the likely cholesterol binding motif in this groove [52] is spatially close to two mutations we have made that resulted in perturbed folding and maturation of ABCG2 (L633A this paper and I573A [21]), suggesting that stabilisation of this site by cholesterol may be essential to maintain the structural integrity of the protein.

Our two-site model for MX binding proposes a lipid exposed (surface) site and a deeper (buried) site, and this has parallels in other transporters. For ABCB1, there are experimental and computational data supporting binding sites for drugs at the lipid:protein interface [7,9,53]. Similarly, the bacterial tripartite multidrug pumps (exemplified by AcrABTolC) are known to have both surface accessible and buried binding sites for the same drug substrate [54–56]. Indeed, it is a parallel to the latter pump that we believe embodies the data we have presented. Namely that despite its inherent 2-fold sequence identity the ABCG2 dimer has, at least, two binding conformations for MX, and that there is structural and functional asymmetry in the ABCG2 dimer. This makes it tempting to speculate that the two monomers cycle between conformations allowing drug binding and drug release upon the alternating hydrolysis of ATP at the two NBDs, in the same way that AcrB monomers, despite their sequence identity, cycle through three different conformations upon proton transport [56]. Whether this is an accurate description of MX transport remains to be elucidated by future studies.

## Abbreviations

DMEM: Dulbecco's Modified Eagle Medium
DNR: daunorubicin
FCS: foetal calf serum
MX: mitoxantrone
PBS: phosphate-buffered saline
PEI: polyethyleneimine
PhA: pheophorbide A
sfGFP: superfolder GFP
TM3: transmembrane helix 3
WT: wild-type
