# Supplementary material for: Residues contributing to drug transport by ABCG2 are localised to multiple drug-binding pockets
Source: Biochem J. 2018 May 4;475(9):1553–67. doi: 10.1042/BCJ20170923 (PMC5934980; doi:10.1042/BCJ20170923)

Supplementary Data

Table S1. List of primers used in this study.

Mutation	Primer 5’-3’
T402A	cagatcattgtc**Gca**gtcgtactgggac
L405A	gtcacagtcgta**GCg**gactggttatag
S440A	ccaaccagtgtttc**GCc**agtgtttcagcc
S443A	cagcagtgtt**Gca**gccgtggaactc
S519A	gatggtggcttat**GcT**gccagttccatg
M523A	tcagccagttcc**GCA**gcactggccatag
L540A	gtagcaacactt**GCc**atgaccatctgt
I543A	cttctcatgacc**GCc**tgttttgtgtttatg
F640A	gtatgattgttatt**GCc**ctcacaattgcctac
I643A	gttattttcctcaca**GCt**gcctacctgaaattg
D477A	ggaaaactgttatct**GCC**ttattacccatgagg
L478A	ctgttatctgat**GCC**ttacccatgaggatg
L479A	ctgttatctgattta**GCa**cccatgaggatg
P480A	gttatctgatttatta**GcG**atgaggatgttacc
M481A	ctgatttattaccc**GCC**aggatgttacc
R482A	gatttattacccatg**GCg**atgttacc
M483A	gatttattacccatgCgA**GCg**ttaccaagtattatatttacc
L484A	cccatgaggatg**GCa**ccaagtattatatttacc
S486A	gaggatgttacca**GCC**attatatttacctg
I487A	gttaccaagt**GCG**atatttacctg
I488A	gttaccaagtatt**GCC**tttacctgtatagtg
F489A	ccaagtattata**GCG**acctgtatagtgtac
T490A	ccaagtattatattC**Gcc**tgtatagtgtacttcatg
C491A	caagtattatatttacc**GCC**atCgtgtacttcatgttagg
I492A	gtattatatttacctgt**GCC**gtgtacttcatg
V493A	cctgtata**gCg**tacttcatgttaggattg
Y494A	cctgtatagtg**GCG**ttcatgttaggattg
F495A	cctctatagtgtac**GCT**atgttaggattg
M496A	gtatagtgtacttc**GCC**ttaggattg

L497A	gtgtacttcatg**GCG**ggattgaagcc
M541A	gcaacacttctc**GCC**accatctg
F545A	catgaccatctgt**GCC**gtgtttatgat
M548A	ctgttttgtgttt**GCC**atgatttttcagg
F571A	cttcagtac**GCC**agcattcca
L633A	gaatcacgtggcc**GCG**gcttgtatgattg
M636A	gccttggcttgt**GCG**attgttattttcc
Mutations are denoted with the single letter amino acid code. All primers show the mutation to alanine in bold, with the base changes required for this in uppercase. Any other uppercase bases denote changes in sequence silent with respect to the codon, but which allowed for screening by restriction enzyme digestion.	

Table S2. Binding site comparison.
Residues proposed to interact with mitoxantrone from this study, from the Hegedus homology model {Laszlo, 2016 #3547} are listed in four tables for easy comparison. Residues shared between predictions are shaded.

Kerr Surface Site

M523	C544	F547	M548	F571
I639	F640			

Kerr Buried Site

Q398	V401	T402	L405	L433
N436	F439	S440	S443	L447
D477	L478	M481	R482	P485
S486	I488	F489	T490	M510
L513	M514	V516	A517	S521

Hegedus Site 2

L388	A394	A397	Q398	I399
V401	T402	L405	Q437	C438
S440	S441	V442	S443	A444
V445	E446	L447	F448	V450
K473	D477	L478	M481	R482
P485	S486	A517	A520	S521
A524				

Hegedus Site 3

A397	V401	F439	S440	V442
S443	V445	E446	L447	V449
V533	V534	S535	V536	A537
T538	L539	L540	M541	T542
I543	F545			

Supplementary Figure Legends

Supplementary Figure S1. Conservation of transmembrane helix 3 in ABCG2. Sequences were aligned using ClustalO and the yellow highlighted area represents residues 477-497 of human ABCG2. Conservation is denoted by "*" and similarity by ":" or "." below the alignment.

Supplementary Figure S2. Cell surface localisation of the ABCG2 variants. sfGFP-tagged variant isoforms of ABCG2 were imaged either using a confocal plate reader (excitation wavelength of 488nm and emission bandpass filter of 525/50 nm), or with a confocal laser scanning microscope (excitation wavelength of 488nm and emission collected at 500-550nm). In both cases, cells were subsequently washed twice with pre-warmed (37°C) phenol-red free HBSS (Hank's Balanced Salt Solution, Sigma Aldrich) immediately prior to imaging. Scale bar represents 20 µm.

Supplementary Figure S3. Expression level of ABCG2 variants. Cells expressing mutant isoforms of ABCG2 were quantified by flow cytometry for cellular sfGFP fluorescence as a measure of total sfGFP-ABCG2 protein expression. Upper panel **A** represents TM3 mutants, lower panel **B** represents lateral slice residues. Data are the mean (± standard error of the mean) of at least 3 independent experiments.

Supplementary Figure S4. Catalytically inactivating mutation abrogate gain of transport function. ABCG2 carrying mutation at F640A and Walker B mutation E211Q showed no Ko143-inhibitable transport of daunorubicin confirming that ATP hydrolysis is required for the gain of daunorubicin transport seen in the single F640A mutant.

Supplementary Figure S5. Structural mapping of residues mutated in this study. Top panel inset represents the ABCG2 homology model [29] with mutations colour coded as in previous figures and stick models of both the proposed surface site and buried site for mitoxantrone shown in larger scale in the main figure. Lower panels represent orthogonal views.

Supplementary Figure 1

TM3, 477-497 (Homo sapiens)

```
Homo sapiens                RVSSYFLGKLLSDLLPMRMLPSIIFTCIVYFMLGLKPKADAFFVMMFTLMMVAY
Gallus gallus               RVSAYFLALMLGDLLPMRTAPAIIFSCITYWMIGFQAIAGRFFFFMLALVMVSY
Salmo salar                 RVSVYFLSKILSDIITLRTIPAIIFSCVAYFMIGFKTTPAAFFIFMFTVTLVAY
Xenopus laevis              RLSAYFFAKLFTDLLPMRTLPSIIFTAVLYFMIGFKATAGAFFIMMFTLTMIAY
Mus musculus                RVSSYFFGKVMSDLLPMRFLPSVIFTCVLYFMLGLKKTVDAFFIMMFTLIMVAY
Macaca mulatta              RVSSYFFGKLLSDLLPMRMLPSIIFTCIVYFMLGLKPVVEAFFIMMFTLMMVAY
Canis lupis familiaris      RVSSYFFGKLLSDLLPMRMLPSIIFTCIIYFLLGLKPVVEAFFIMMFTLMMVAY
Sus scrofa                  RVSSYFFGKLLSDLLPMRMLPSIIFTCITYFLLGLKPAVGSFFIMMFTLMMVAY
Bos taurus                  RVSSYFFGKLLSDLLPMRMLPSIIFTCITYFLLGLKPKVEAFFIMMLTLMMVAY
                            *:* **:. :: *:: :*  *::**:.: *:::*::     **.:*::: :::*
```

Supplementary Figure 2

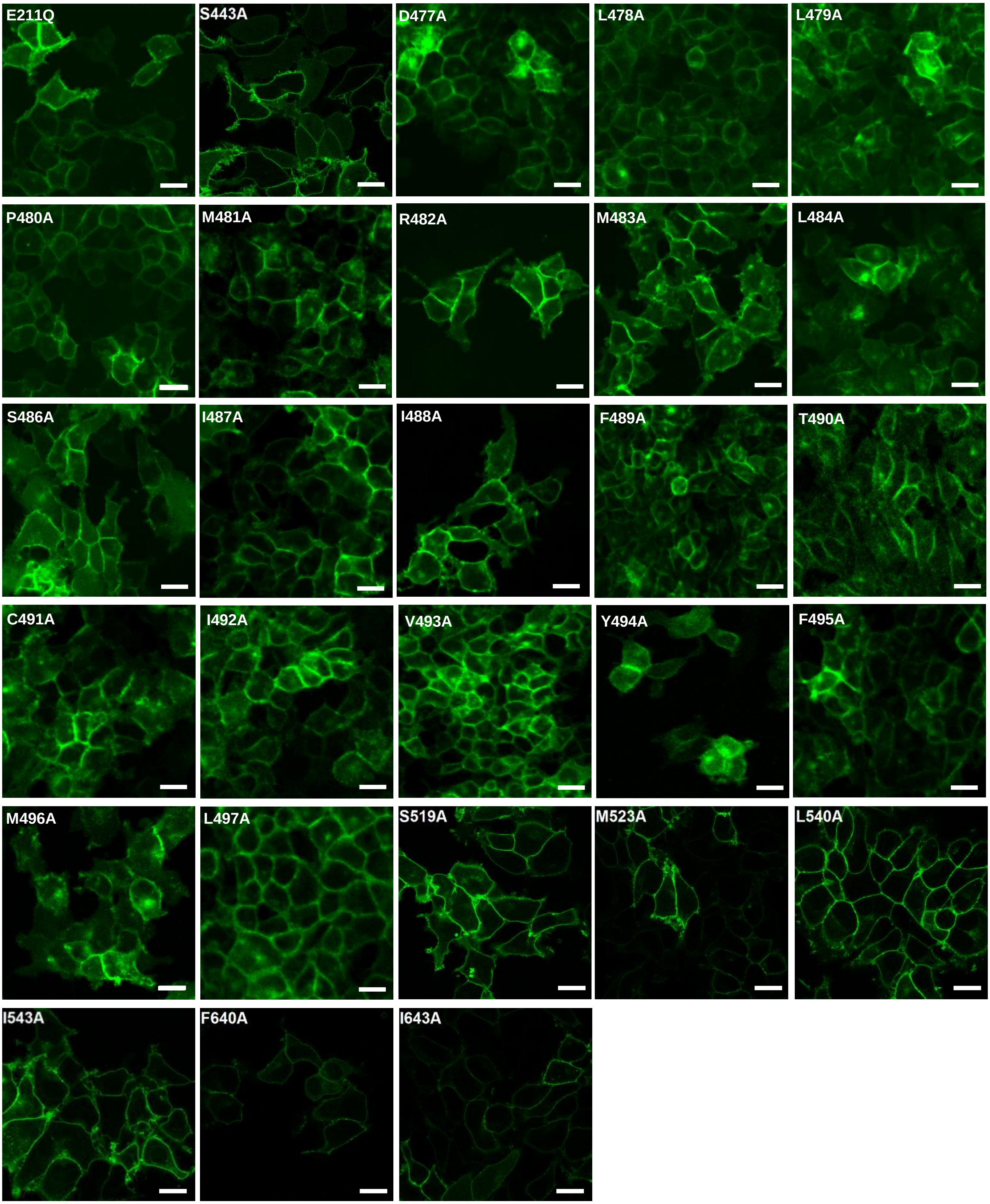

Supplementary Figure 3

A

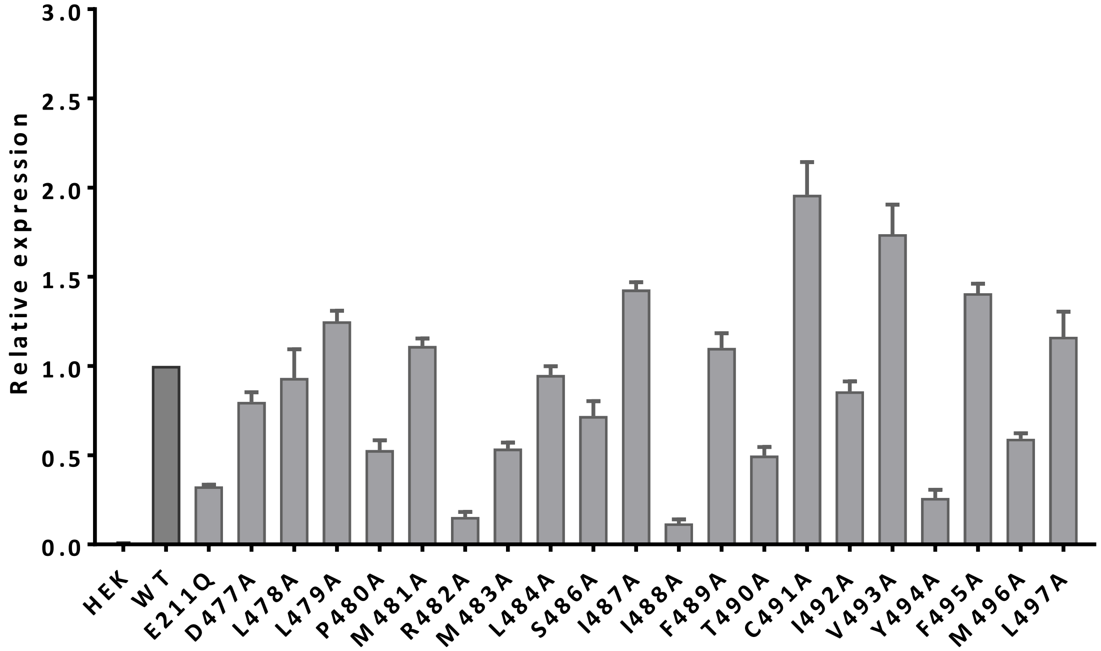

B

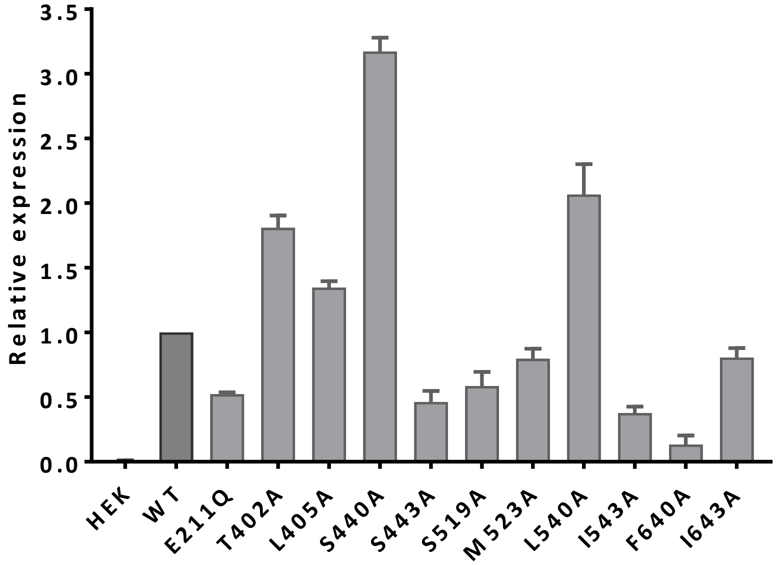

Supplementary Figure 4

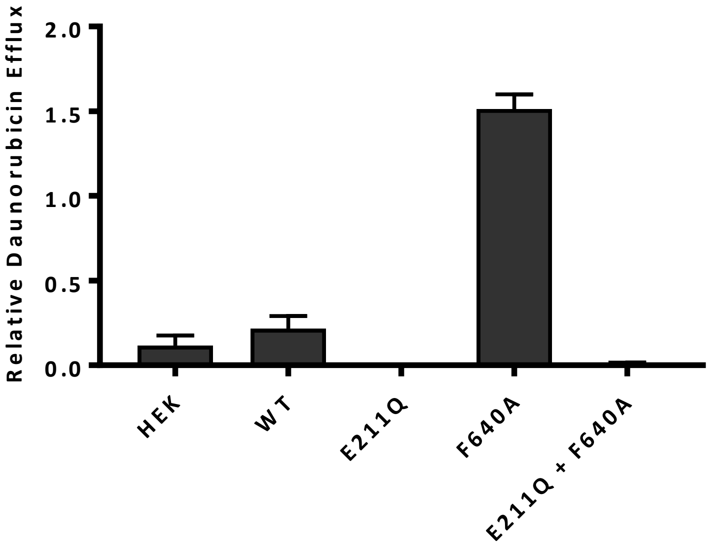

Supplementary Figure 5

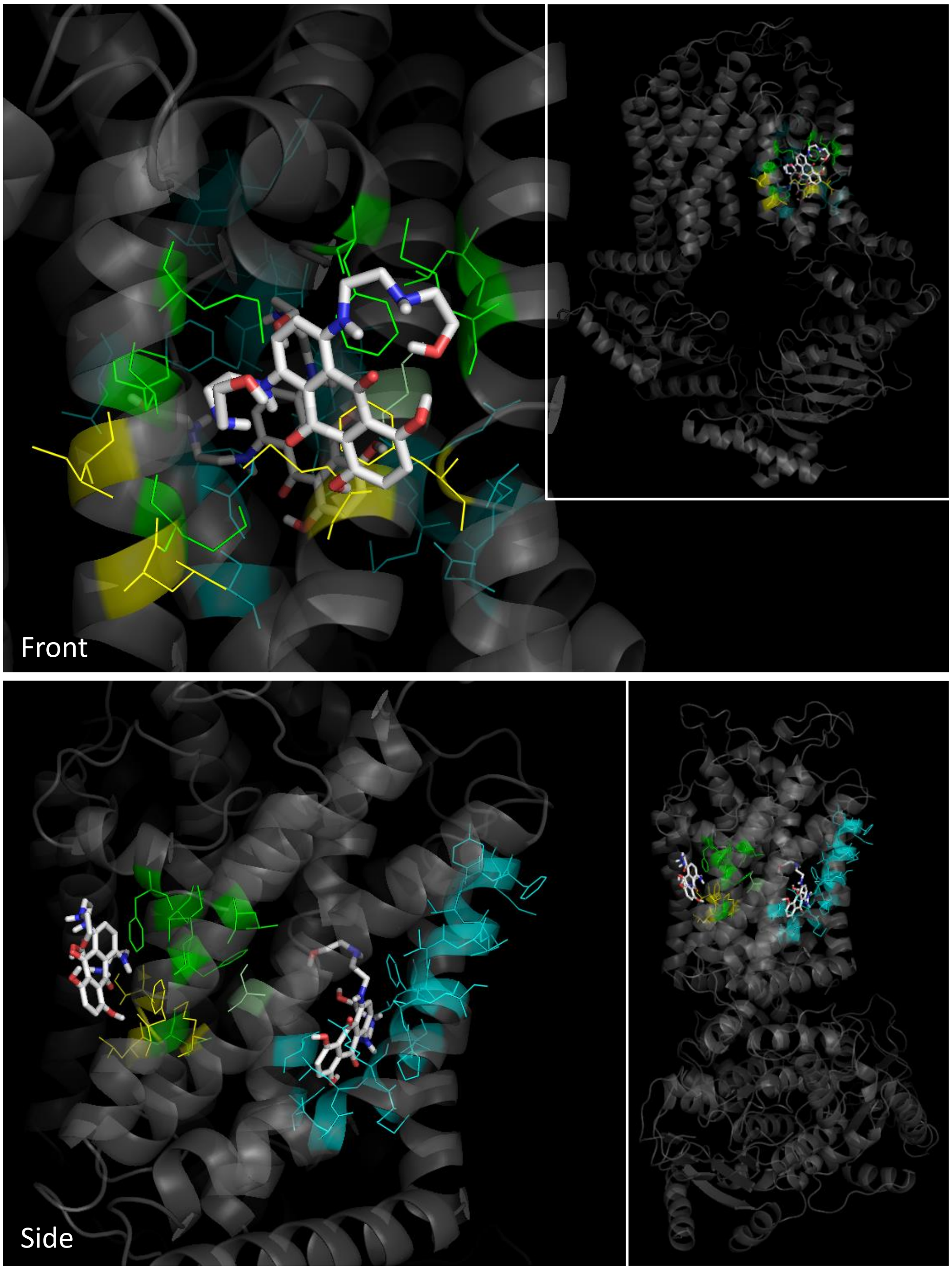

Supplement: Supplementary Tables and Figures [file BCJ-475-1553-s1.pdf]
